# High-Fat Diet Induces Hepatic Insulin Resistance and Impairment of Synaptic Plasticity

**DOI:** 10.1371/journal.pone.0128274

**Published:** 2015-05-29

**Authors:** Zhigang Liu, Ishan Y. Patil, Tianyi Jiang, Harsh Sancheti, John P. Walsh, Bangyan L. Stiles, Fei Yin, Enrique Cadenas

**Affiliations:** 1 Pharmacology & Pharmaceutical Sciences, School of Pharmacy, University of Southern California, Los Angeles, CA, 90089, United States of America; 2 College of Food Science and Engineering, Northwest A&F University, Yangling, China; 3 Davis School of Gerontology and Program in Neuroscience, University of Southern California, Los Angeles, CA, 90089, United States of America; University of Cordoba, SPAIN

## Abstract

High-fat diet (HFD)-induced obesity is associated with insulin resistance, which may affect brain synaptic plasticity through impairment of insulin-sensitive processes underlying neuronal survival, learning, and memory. The experimental model consisted of 3 month-old C57BL/6J mice fed either a normal chow diet (control group) or a HFD (60% of calorie from fat; HFD group) for 12 weeks. This model was characterized as a function of time in terms of body weight, fasting blood glucose and insulin levels, HOMA-IR values, and plasma triglycerides. IRS-1/Akt pathway was assessed in primary hepatocytes and brain homogenates. The effect of HFD in brain was assessed by electrophysiology, input/output responses and long-term potentiation. HFD-fed mice exhibited a significant increase in body weight, higher fasting glucose- and insulin levels in plasma, lower glucose tolerance, and higher HOMA-IR values. In liver, HFD elicited (a) a significant decrease of insulin receptor substrate (IRS-1) phosphorylation on Tyr608 and increase of Ser307 phosphorylation, indicative of IRS-1 inactivation; (b) these changes were accompanied by inflammatory responses in terms of increases in the expression of NFκB and iNOS and activation of the MAP kinases p38 and JNK; (c) primary hepatocytes from mice fed a HFD showed decreased cellular oxygen consumption rates (indicative of mitochondrial functional impairment); this can be ascribed partly to a decreased expression of PGC1**α** and mitochondrial biogenesis. In brain, HFD feeding elicited (a) an inactivation of the IRS-1 and, consequentially, (b) a decreased expression and plasma membrane localization of the insulin-sensitive neuronal glucose transporters GLUT3/GLUT4; (c) a suppression of the ERK/CREB pathway, and (d) a substantial decrease in long-term potentiation in the CA1 region of hippocampus (indicative of impaired synaptic plasticity). It may be surmised that 12 weeks fed with HFD induce a systemic insulin resistance that impacts profoundly on brain activity, i.e., synaptic plasticity.

## Introduction

Nutritional overload in the form of high dietary intake of fats—modulated by genetic and environmental factors—is associated with a number of somatic disorders, such as obesity, type 2 diabetes mellitus, cardiovascular diseases, and metabolic syndrome. Elevated triglycerides, blood pressure, and fasting glucose, and reduced HDL cholesterol are recognized as major risk factors for these disorders. Moreover, these disorders share a common pathological condition, insulin resistance, which entails a progressive reduction in the responsiveness of peripheral tissue to insulin due to nutritional overload, chronic inflammation, dyslipidemia, and hyperglycemia. Clinically, insulin resistance may be manifested by glucose intolerance for years before the diagnosis of diabetes, due to the effort of the endocrine pancreas to increase insulin secretion to maintain normal glucose levels.

In the peripheral tissues, insulin resistance is associated with compromised cell metabolism and survival, increased oxidative stress and activated inflammatory responses (cytokine activation). Insulin resistance entails disruption of metabolic homeostasis, largely due to mitochondrial dysfunction; this impairs cellular function at multiple levels with a broad range of consequences, from increased oxidative stress, DNA damage, to several forms of cell death [1, 2]. In liver, insulin resistance is a factor of the progression of non-alcoholic fatty liver disease (NAFLD) [3, 4]. The latter progresses with hyperinsulinemia and inhibition of the insulin receptor substrate (IRS) [5, 6].

In addition to the peripheral effects, insulin plays important roles in the central nervous system. Insulin receptor is expressed throughout the brain but shows higher levels in metabolically active regions such as hippocampus, cerebral cortex, hypothalamus, and cerebellum [7]. Insulin is implicated in neuronal survival, synaptic plasticity, memory and learning [8, 9] primarily due to its role in initiating two canonical pathways downstream of the insulin receptor: IRS-PI3K-Akt pathway and the Shc-Ras-ERK pathway [10]. Activation of the PI3K-Akt pathway induces the expression of insulin-sensitive glucose transporters (GLUT4) and promote energy metabolism and cell survival, whereas the Ras-ERK pathway modulates the expression of genes involved in synaptic plasticity and cell differentiation [11].

Insulin resistance in brain is associated with synaptodendritic abnormalities and memory disorder [12, 13], impaired hippocampal neurogenesis [14, 15], decreased the expression of BDNF^15^, and diminished cognitive performance [16–18]. Systemic insulin resistance and defective brain insulin signaling are common features of Alzheimer’s disease [19], thus rendering type 2 diabetes an important risk factor for this neurodegenerative disorder. Epidemiological studies indicate that long-term hyperinsulinemia is also a risk factor for dementia [20, 21], whereas insulin administration to healthy individuals, by keeping glucose levels constant, improves memory formation [22]. The underlying mechanism of how perturbations in peripheral environment affect brain function is currently unclear. It was proposed that a liver-brain axis contribute to the connection, whereby toxic lipids produced in liver cross the blood brain barrier and cause brain insulin resistance [17, 23, 24].

Therefore, the significance of systemic environment in liver and brain function need to be recognized and explored by determining whether, and if so how, diet-induced systemic insulin resistance influences energy metabolism and synaptic transmission in the brain. This study is aimed at establishing such a connection by assessing the effects of systemic insulin resistance on liver and brain metabolic homeostasis, inflammatory responses, and synaptic plasticity using a high fat diet-induced obesity rodent model.

## Materials and Methods

### Animals and Diet

3 month-old C57BL/6J mice were purchased from Jackson Laboratories (Sacramento, CA, USA). Mice were housed in the animal facility under standard conditions (12/12 light-dark cycle, humidity at 50 ± 15%, temperature 22 ± 2°C) and assigned to two groups (*n* = 10/group) fed with standard diet (5053 Labdiet, 13% kcal from fat; diet composition: protein 20%, fat 5%, fiber 4.7%, ash 6.1%, minerals 3.03%) or high-fat diet (Harlan Teklad TD.06414, 60% kcal from fat; diet composition: protein 23.5%, fat 27.3%, carbohydrate 27.3%, mineral mix: AIN-93G-MX(94046) 4.8%) for 12 weeks. Body weight and food intake were recorded weekly.

All surgery including orbital eye bleeding and euthanasia were performed under anesthesia by continuously inhalation of 2% isoflurane via a nose cone. All procedures were approved by USC Department of Animal Resources and the Institutional Animal Care and Use Committee (protocol number: 11211).

### Serum assays

Assays were performed as previously described [25]; fasting glucose was determined from overnight-fasted mice. For glucose tolerance test (after 12 weeks feeding), glucose (1 g/kg body weight) was injected intraperitoneally and blood glucose was measured with an OneTouch Ultra glucometer (Lifescan Benelux, Beerse, Belgium) before and 5, 15, 30, 60, and 120 min after the injection. Serum separated from orbital eye bleeding was determined for insulin levels with an insulin Elisa kit (Alpco, Salem, NH, USA)

### Isolation of hepatocytes

Hepatocytes were isolated as previously described [26]. Briefly, the liver was perfused with collagenase and isolated hepatocytes (viability >90%) were plated in individual 60-mm-diameter LUX culture dishes or in Seahorse XF-24 (Seahorse BioSciences, Billerica, MA, USA) plates pre-coated with 0.03% rat tail collagen. After 3 h, the culture medium was changed to serum-free medium containing 100 U/mL penicillin and 0.1 mg/mL streptomycin for further analysis.

### Metabolic flux analysis

Metabolic flux analysis was performed as described in [27]: briefly, primary hepatocytes media was changed to unbuffered DMEM and incubated at 37°C in a non-CO_2_ incubator for 1 h using the XF-24 Extraflux Analyzer (Seahorse BioSciences). Three baseline measurements of OCR (Oxygen Consumption Rate) were sampled prior to sequential injection of mitochondrial inhibitors: oligomycin (4 μM), FCCP (0.2 μM), and rotenone (1 μM). Oxygen consumption rates were automatically calculated and recorded by the Seahorse XF-24 software and normalized to protein level in each well.

### Brain protein preparation

Whole brain homogenates were prepared using T-PER (Pierce Biotechnology, Rockford, IL, USA) and the cytosolic and nuclear fractions were isolated using NE-PERTM Nuclear and Cytoplasmic Extraction Reagents (Pierce Biotechnology, Rockford, IL, USA).

### Western blot analyses

Hepatocyte lysate, brain homogenates, membrane and nuclear extractions were solubilized in SDS sample buffer, separated by Laemmli SDS/PAGE, and transferred onto PVDF membranes. Using appropriate antibodies (dilution rate of primary antibodies is1:1000, secondary antibodies is 1:2000), the immuno-reactive bands were visualized with an enhanced chemiluminescence reagent. The primary antibodies against IRS-1 (C-20), **β**-actin (SC-1616), GLUT3 (sc-74497), GLUT4 (sc-1608), Na^+^/K^+^-ATPase (sc-58628), ERK (sc-292838) and HRP-labeled secondary antibodies were obtained from Santa Cruz Biotechnology (Dallas, Texas, USA). Akt (9272), p-Akt (Ser473) (9271), PGC1**α** (2178), JNK1/2 (9258), p-JNK (9255), p38 (8690), p-p38 (9211), NF**κ**B (8242), iNOS (13120), p-c-Raf (9431), p-ERK (4370), p-CREB (9196) and CREB (820) antibodies were got from Cell Signaling (Danvers, MA, USA). p-IRS1 (Tyr608) (09–432) were got from Millipore (Billerica, MA, USA).

### Long-Term Potentiation and I/O Curves

Electrophysiology experiments were performed as described in [28]. Each animal was decapitated and the brain was rapidly removed and immersed in sucrose-modified artificial cerebrospinal fluid (aCSF) and was cut to contain the hippocampus and coronal 350 μm thick hippocampal slices with surrounding cortical tissue using a vibratome (Series 1000, St Louis, MO, USA) after 3-min cooling. Slices were transferred to chamber with oxygenated aCSF at room temperature. After 1 h of equilibrium, one slice was transferred to an interface recording chamber and perfused with aCSF, with the surface of slices exposed to warm, humidified 95% O_2_-5% CO_2_. Field EPSPs (fEPSPs) were recorded from stratum radiatum of CA1 using a glass pipette filled with 2M NaCl in response to orthodromic stimulation of the Schaffer collateral-commissural projections. Pulses of 0.1 ms duration were delivered to the stimulating electrode every 20 s. The responses were amplified with Axoclamp 2A DC amplifier; data acquisition was controlled by Clampex 9.0 software (Axon Instruments, Foster City, CA, USA). Input/output (I/O) curves were generated using stimulus intensities from 100–450 μA in increments of 50 μA. Baseline fEPSP were evoked at 30–50% of maximal fEPSP in 20 s intervals. Long-term potentiation (LTP) was induced at baseline intensity using Theta Burst Stimulation (TBS) consisting of ten trains of five 100 Hz stimulation repeated at 5 Hz. Recording continued for at least 30 min following TBS. fEPSP slope magnitude was calculated as the difference between two cursors, separated by 1 ms. LTP values were expressed as a percentage of the average slope from the baseline recordings. The last 5 min of responses to TBS stimulator were analyzed.

### Data analysis

Data are reported as means ± SD of at least three independent experiments. Significant differences between mean values were determined by Student t-test. Means were considered to be statistically distinct if *p* < 0.05.

## Results

### Properties of the HFD-induced obesity model of C57BL/6J mice

**Fig 1:**
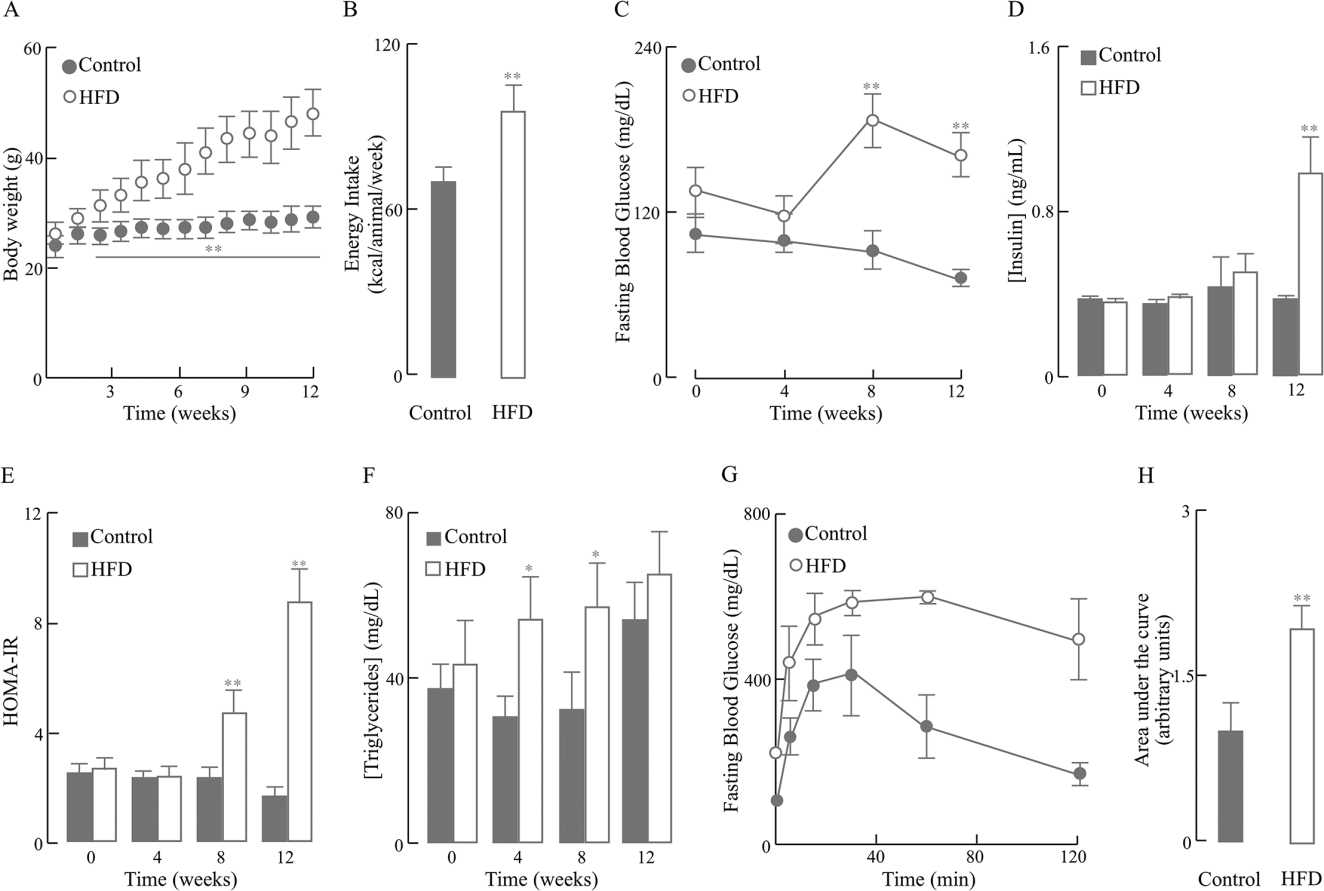
Characterization of the HFD-induced mouse model of obesity. Mice were fed with 12-week HFD or normal diet and different parameters were monitored weekly or monthly. (A) Body weight; (B) Energy intake; (C) Fasting blood glucose concentration; (D) Fasting insulin concentration; (E) Insulin resistance index, HOMA-IR; (F) Fasting triglyceride levels; (G) Glucose tolerance test at 12 week; (H) Area under curve (AUC) analyses for glucose tolerance tests. Data presented as mean ± SD, n ≥ 3 mice/group, *p < 0.05, **p < 0.01 *versus* control group.

To establish a mouse model of HFD-induced obesity, 3 month-old male C57BL/6J mice were randomized into two groups (*n* = 10 per group) fed with either control diet or HFD for 12 weeks. Their body weight and food intake were monitored. Key parameters related to obesity and insulin resistance were measured on a monthly basis. Compared to the control group, HFD led to elevated body weight starting from week 3 (20.5% higher than control group) throughout the feeding period (62.5% higher than control group at week 12) (Fig 1A). Increased body weight in HFD group was accompanied by a 27.7% increase in their weekly calorie intake compared to control group (Fig 1B).

As expected, hyperglycemia was developed after 8-week HFD feeding and persisted through week 12, as shown by 92.8% and 109.8% higher circulating glucose levels than the control group at these two time points, respectively (Fig 1C). In contrast, hyperinsulinemia did not develop until week 12 in the HFD mice; at this time point, the circulating insulin in the HFD group was 72% higher than that in the control group (Fig 1D). These changes in circulating glucose and insulin levels induced by HFD were further reflected in a significant increase in the HOMA-IR index, a quantitative measure of insulin resistance, which appeared at week 8 and augmented at week 12, although no change was seen in earlier time points (Fig 1E). Consistently, in the HFD-fed mice at week 12, insulin resistance—as indicated by a 5.3-fold increase in HOMA-IR index relative to control group—culminated in glucose intolerance (Fig 1G and 1H), which was observed as early as week 4 (S1 Fig Glucose Tolerance Test and Liver Weight). Interestingly, statistically significant increased triglyceride levels in HFD group were observed at week 4 and 8 time points but not at week 12 (Fig 1F); this was largely due to a significant increase of triglyceride levels in the control mice at week 12 compared to their levels at week 8. In addition to the parameters shown in Fig 1, liver weight increased by ~76% at 12 weeks of HFD feeding (S1 Fig Glucose Tolerance Test and Liver Weight).

### Effect of HFD on energy metabolism and inflammatory responses in liver

**Fig 2:**
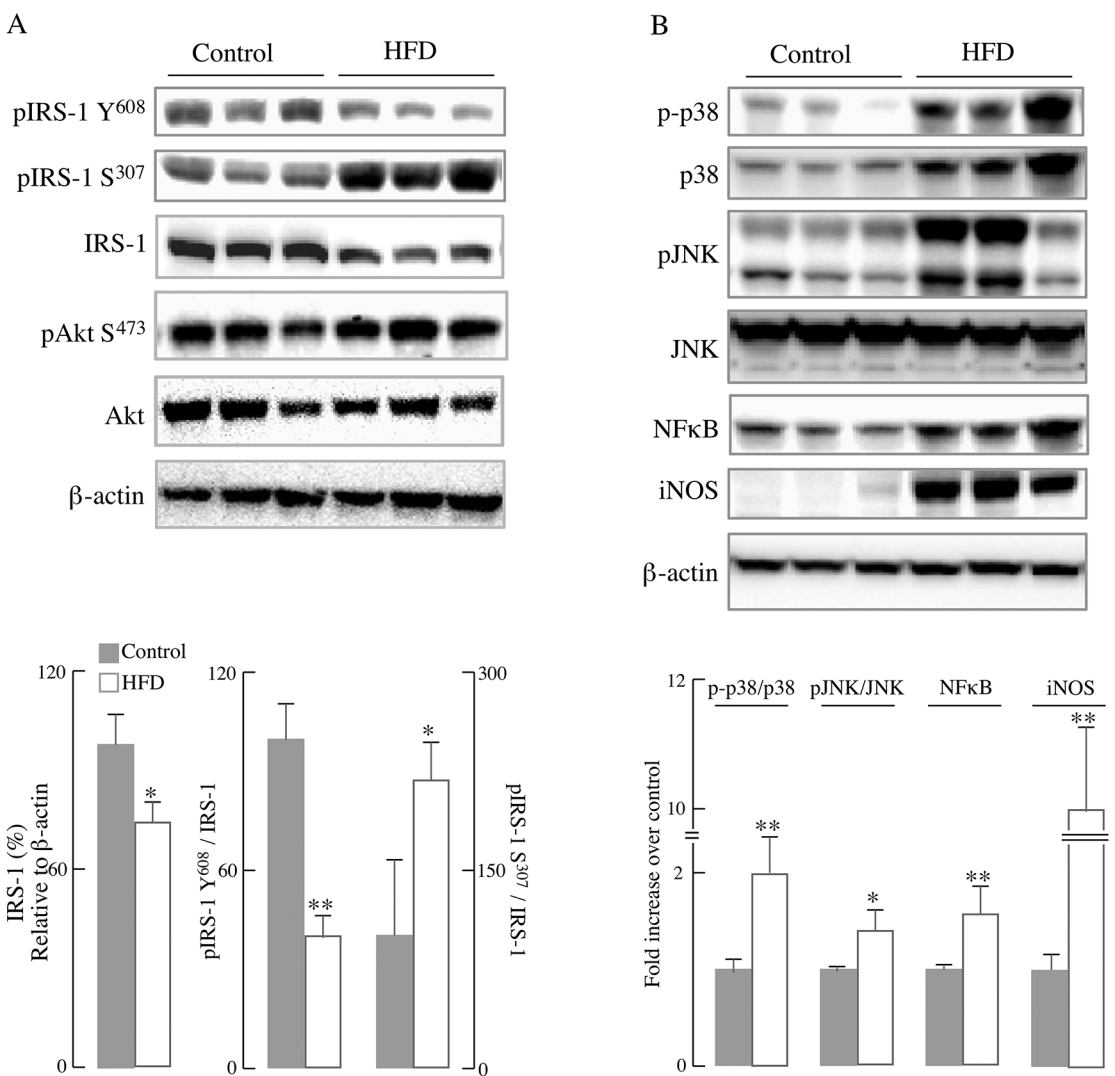
HFD led to impairment of IRS-1/Akt pathway and activation of inflammatory responses in primary hepatocytes. Primary hepatocytes were isolated from mice fed a normal diet or HFD for 12 weeks (as described in the Materials and Methods section). (A) Representative western blots of IRS-1/Akt pathway proteins and the respective densitometry measurements; (B) Representative western blots of inflammatory proteins and the corresponding densitometry measurements. Data presented as mean ± SD, *n* ≥3 mice/group, *p < 0.05, ** *p* < 0.01 *versus* control group.

To assess the impact of HFD on liver metabolic function and inflammatory responses, primary hepatocytes were isolated from mice fed a normal diet or HFD for 12 weeks. In line with the insulin resistance shown in Fig 1E, IRS-1/Akt pathway in primary hepatocytes isolated from HFD-fed mice was substantially decreased as compared with the control group: there was a marked decrease in total IRS-1 (24% decline, *p* < 0.01) as well as the phosphorylated IRS-1 at Tyr^608^ (60.2% decline, *p* < 0.01) in mice fed with HFD compared with those on a control diet (Fig 2A), whereas there was a 120.3% increase (*p* < 0.05) in the phosphorylated IRS-1 at Ser^307^. IRS-1 phosphorylation at Tyr^608^ is associated with its activation, whereas phosphorylation at Ser^307^ is associated with inactivation of IRS-1; phosphorylation of IRS-1 at Ser^307^ is likely accomplished by JNK [29]. Despite the pronounced decrease in IRS activation (pIRS-1-Y^608^) in the HFD group, there were no significant changes in the levels of Akt phosphorylated at Ser^473^ (Fig 2A).

Chronic inflammation is associated with obesity-induced insulin resistance, largely by inhibiting the insulin pathway at IRS-1 [30]. IRS-1 can be phosphorylated and inhibited at its serine residues by the stress-sensitive JNK pathway [29] or the inflammation-regulating NFκB pathway [31]. The data indicated an activation of the major components of the inflammatory pathways in primary hepatocytes isolated from mice fed a HFD for 12 weeks (Fig 2B): the activation (phosphorylation) of both MAPKs, p38 and JNK, was substantially increased as compared with the control group. NF**κ**B, which plays a central stage in inflammatory responses, exhibited a higher expression in HFD group; a transcriptional target of NF**κ**B, the inducible nitric oxide synthase (iNOS) was increased over 10-fold in primary hepatocytes from HFD-fed mice (Fig 2B).

**Fig 3:**
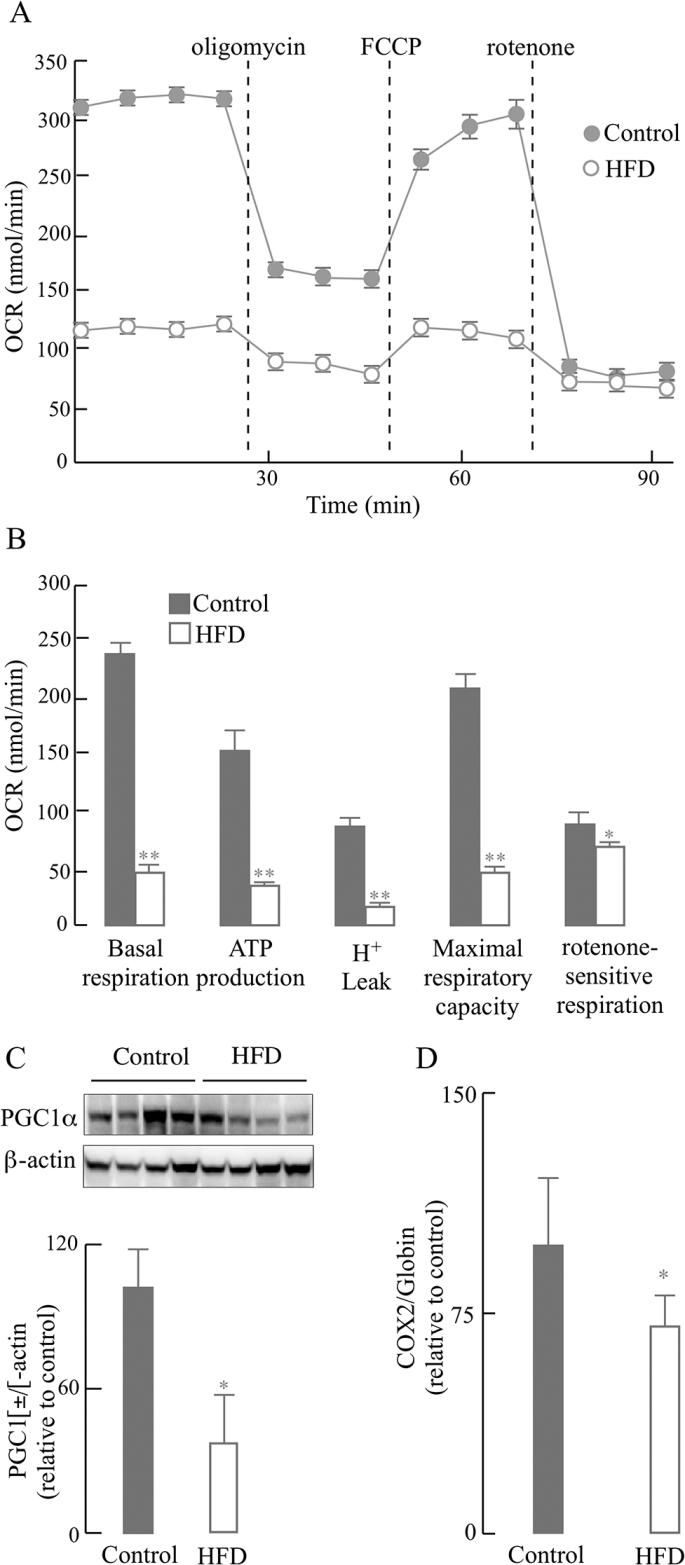
HFD-induced mitochondria dysfunction in primary hepatocytes. Primary hepatocytes were isolated from mice fed a normal diet or HFD for 12 weeks as described in the Materials and Methods section. (A) Time course of Oxygen Consumption Rate (OCR), determined using XF-24 Extraflux Analyzer as described in the Materials and Methods section. (B) Quantification of OCR (bioenergetic) parameters in primary hepatocytes (from data in Fig 3A). (C) Effect of HFD on PGC1α expression (western blots and the corresponding densitometry analysis). (D) Effect of HFD on mitochondrial biogenesis (expressed as the ratio of COX2 (mitochondrial DNA) to globin (nuclear DNA)), measured by real-time PCR with specific primers. Data presented as mean ± SD, *n* ≥ 3 mice/group, *p < 0.05, **p < 0.01 *versus* control group.

Chronic inflammation and inhibited insulin pathway are usually associated with impairment of energy metabolism in the liver. Therefore, mitochondrial energy metabolism (OCR) in primary hepatocytes isolated from the HFD-fed and control mice was assessed by the extra-flux analyzer (Fig 3A): the decrease in basal respiration after addition of oligomycin served as an indicator for ATP production and the further addition of FCCP to uncouple mitochondrial respiration from oxidative phosphorylation yielded the maximal respiration. The difference between maximal respiration rate and basal respiration is indicated as the spare respiratory capacity. Primary hepatocytes from HFD-fed mice showed a substantial decrease in baseline respiration as well as in ATP-linked respiration (oxidative phosphorylation) and their response to the uncoupler FCCP was also decreased. Primary hepatocytes from HFD-fed and control mice did not show any reserve capacity despite different concentrations of FCCP tested. The effects of HFD on energy parameters related to OCR are summarized in Fig 3B: HFD decreased basal respiration, ATP-linked respiration, H^+^ leak-induced O_2_ consumption, maximal respiratory capacity, and rotenone-sensitive respiration by 79.4%, 78.7%, 81.0%, 78.0%, and 12.4%, respectively (*p* < 0.05). The latter, rotenone-sensitive respiration includes both complex II-driven O_2_ consumption and non-mitochondrial O_2_ consumption. The decreased mitochondrial respiratory capacity in HFD hepatocytes was partially due to the decline in mitochondria number, as indicated by the lower ratio of mitochondrial DNA (mtDNA) to nuclear DNA (nDNA) (Fig 3D); accordingly, the expression of the master regulator of the mitochondrial biogenesis, PGC1α, was significantly reduced in the HFD group (Fig 3C).

### Effects of HFD on brain

**Fig 4:**
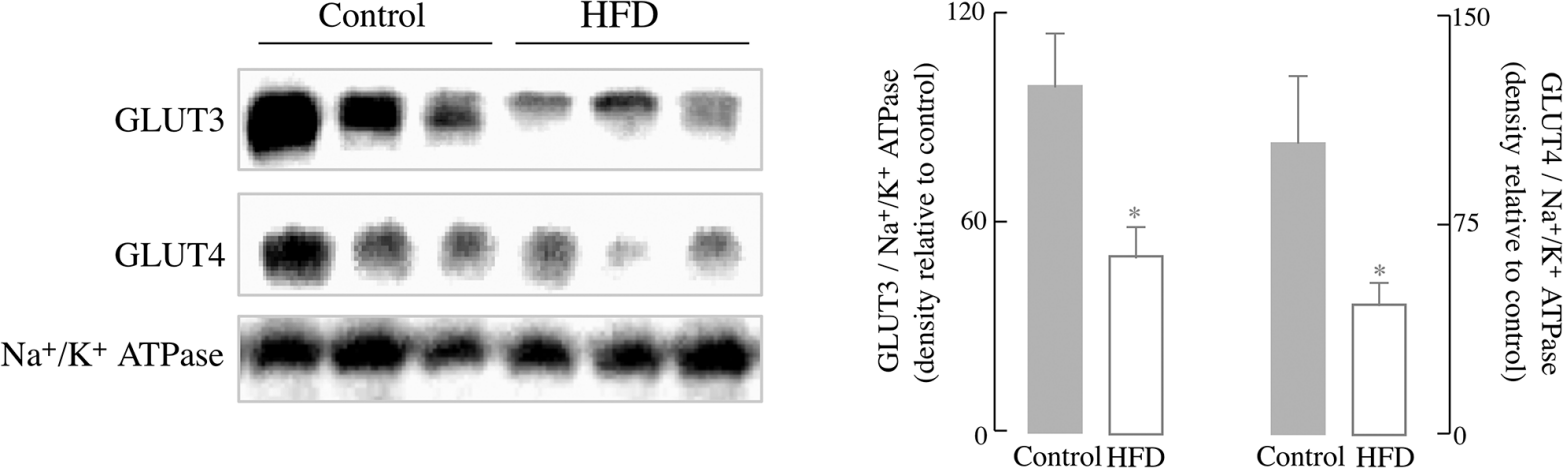
HFD led to a decrease in the levels of membrane-associated glucose transporters. Representative western blots of expression of neuronal glucose transporter 3 (GLUT3) and neuronal glucose transporter 4 (GLUT4). Quantification against a control for total membrane (Na^+^/K^+^ ATPase). Data presented as mean ± SD, *n* ≥ 3 mice/group, *p < 0.05 *versus* control group.

The role of insulin in the central nervous system and its involvement in neuronal survival and plasticity are mediated by a signal transduction cascade identified as the PI3K/Akt route, a major integrator of insulin signaling in brain [9]. The brain energy demands are largely met by glucose uptake and metabolism and, secondarily, by ketone bodies. The neuronal glucose transporter 3 (GLUT3) and glucose transporter 4 (GLUT4) levels in the membrane fraction of the brain tissue indicate the amount of the functional forms of these transporters. The effect of HFD on these neuronal glucose transporters in the brain is shown in Fig 4: plasma membrane-located GLUT3 decreased by 49.0% (*p* < 0.05) and GLUT4 decreased by 55.1% (*p* < 0.05) in brains of mice fed with HFD (compared to controls) (Fig 4). These data indicate an impaired glucose uptake in the brain after HFD.

**Fig 5:**
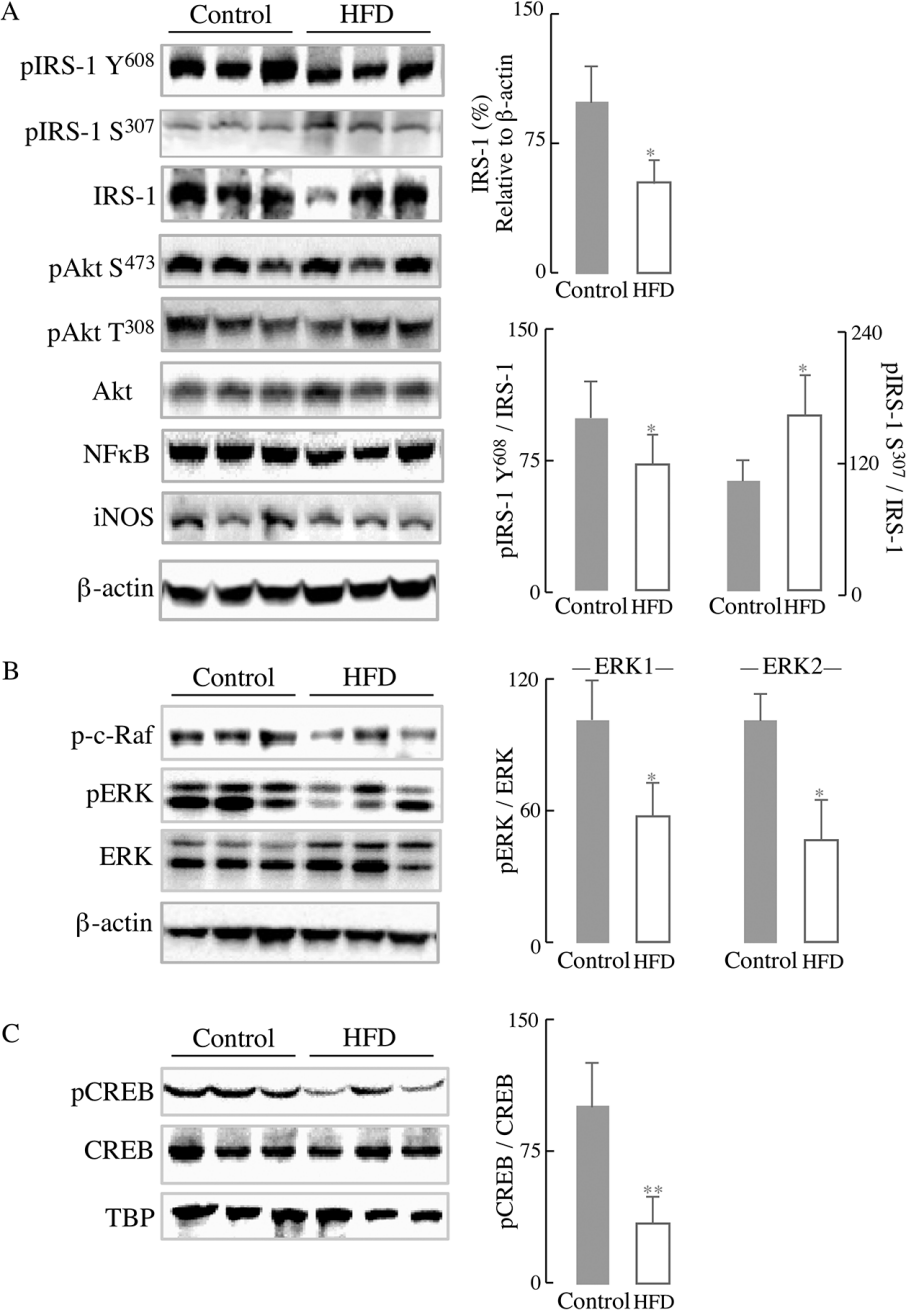
Effect of HFD on insulin signaling: IRS and ERK/CREB pathways. (A) Western blots of the expression of phosphorylated IRS-1, total IRS-1, phosphorylated Akt, and total Akt. Densitometry analyses on the right of panel A. (B) Western blots pERK and ERK expression. Densitometry analysis on the right of panel B. (C) Western blots of pCREB and CREB and densitometry analysis on the right. Whole brain homogenates were used for (A) and (B) and nuclei extraction fractions for (C). Data presented as mean ± SD, *n* ≥ 3 mice/group, *p < 0.05, ** *p* < 0.01 *versus* control group.

Insulin signaling can induce both the expression of GLUT3/4 and their translocation to the plasma membrane. In line with the reduction of GLUT3/4 concentration to the plasma membrane, the levels of total IRS-1 and its phosphorylation at Tyr^608^/Ser^307^ in HFD mice brain were altered and showed the same trend as in liver (Fig 5A). There was a marked decrease in total IRS-1 (48.3%, *p* < 0.05) and the phosphorylation of IRS-1 at Tyr^608^ (36.2%, *p* < 0.01) in mice fed with HFD compared with controls (Fig 5A); on the other hand, there was a significant increase of IRS-1 phosphorylated at Ser^307^ (55.1%, *p* < 0.05), suggesting an inactivation of IRS-1 in the brain of HFD-fed mice (Fig 5A). Similar to what observed in the liver, there was no significant difference in Akt activation (both at Ser^473^ and Thr^308^) between the two groups, after fasting overnight without extra insulin stimulation. At variance with liver, no inflammatory responses—lack of increase in the expression of NFκB and iNOS—were observed in brain. It may be surmised that insulin signaling—a main regulator of metabolism and function in the brain—is impaired in this 12-week feeding mode.

In addition to the PI3K/Akt route, activation of the insulin receptor is associated with activation of effector proteins Ras/Raf/ERK, an insulin-signaling node that is implicated in multiple forms of brain synaptic plasticity [9, 32]. Following activation of the insulin receptor, the Ras guanine nucleotide exchange factor facilitates the recruitment of the kinase Raf, which in turn leads to the phosphorylation of ERK1/2 on threonine and tyrosine residues. ERK also mediates the phosphorylation of the cAMP-response element binding protein (CREB) [33], which is essential for several forms of learning and memory [34]. The phosphorylation of c-Raf was significantly reduced (by 26.2%, *p* < 0.05) in the brain of HFD-fed mice. Consequently, the activation of ERK1/2 (pERK/ERK) was also reduced (pERK1/ERK1 by 43.8% and pERK2/ERK2 by 53.3%; *p* < 0.05) in the HFD group. The downstream CREB activation (pCREB/CREB) was decreased by 65.8% (*p* < 0.01) in the HFD group (Fig 5C).

**Fig 6:**
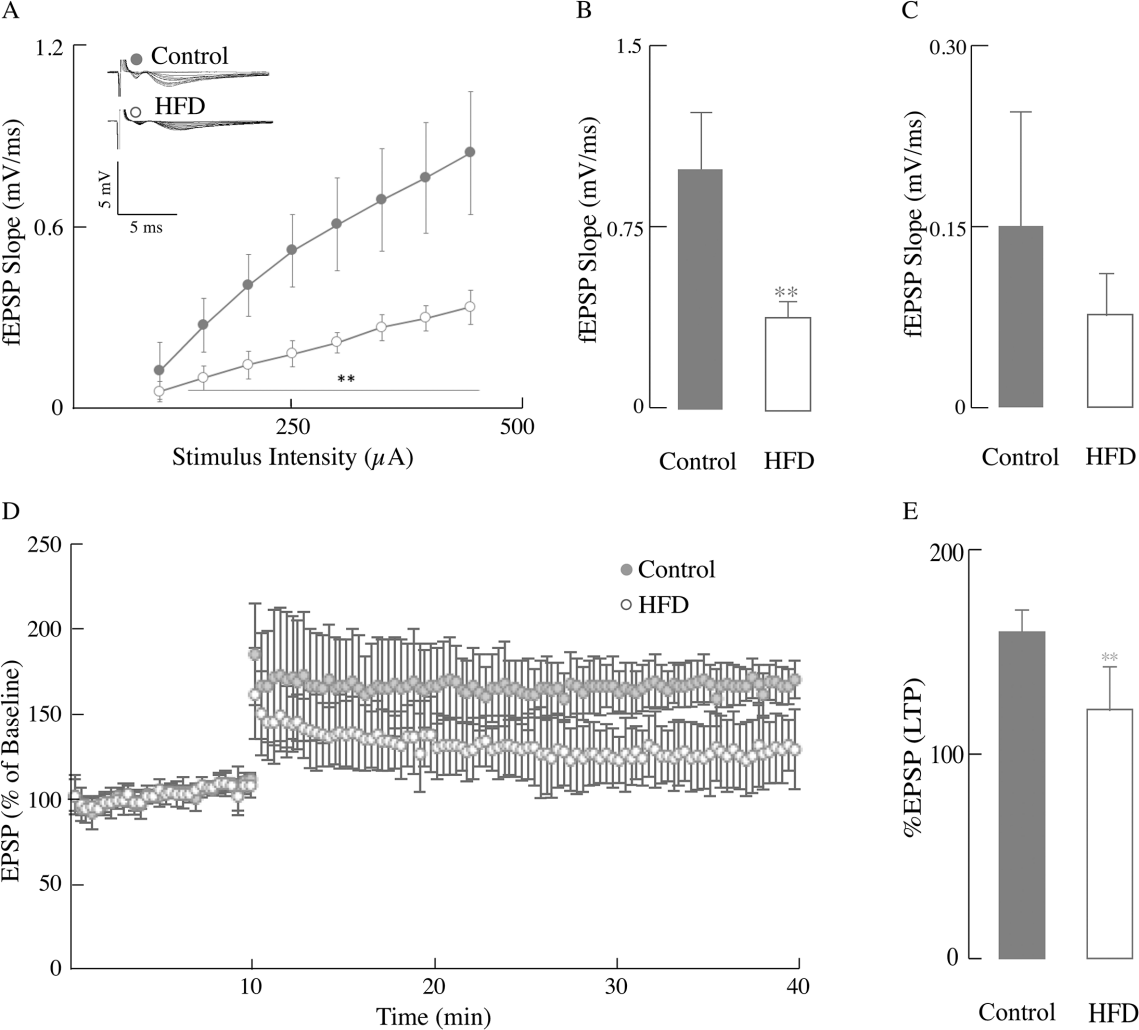
HFD induced compromised hippocampal synaptic plasticity. Input/Output (I/O) and LTP changes were measured in mice fed with normal chow or HFD. (A) I/O relationships after applying increasing stimulation to the stratum radiatum of the CA1 region in the hippocampus for control group mice and HFD group mice and recording the output (electrophysiology as described in the Materials and Methods section). fEPSP slope (mV/ms) plotted against the corresponding stimulation intensity of HFD group (open circles) and control group (close circles). Bar graphs showing the maximum (B) and minimum (C) fEPSP slope values at 400 μA, *n* ≥ 6 slices/group and at least 3–4 animals/group. (D) LTP was induced at baseline intensity using theta burst stimulation (TBS) consisting of ten trains of five 100 Hz stimulation repeated at 5 Hz. Slope of EPSPs was measured and results normalized to the average value measured during the 10 min baseline period. Recording continued for at least 30 min following TBS and the last 5 min was used to calculate the LTP. It shows the first 10 min of baseline followed by the percentage of the baseline response elicited after TBS for 30 min for control and HFD mice. (E) Bar graphs showing the measured LTP using % EPSP for the last 5 min of the response to TBS stimulation for control and HFD mice. Total *n* = 20 slices, *n* ≥ 9 slices/group and at least 3–4 animals/group. Data presented as mean ± SD, *p < 0.05, **p < 0.01 *versus* control group.

The levels of NMDA receptor and perhaps the AMPA receptor recycling are influenced by insulin; these molecular effects of insulin are thus thought to play a pivotal role in its ability to regulate synaptic plasticity via modulation of LTP and long-term depression (LTD) [9]. The association of impaired insulin signaling (from the IRS-1 node to the Ras/Raf/ERK cascade) (Fig 5) with synaptic plasticity was assessed by electrophysiology to measure the Input/Output (I/O) responses, which are indicative of the strength of synaptic plasticity or the connections between neurons. HFD-fed mice had a substantially reduced I/O response in the CA1 region of hippocampus compared to control mice (Fig 6A). In the HFD mice, the maximum output (Fig 6B) was significantly decreased (60.9%, *p* < 0.01) as compared with the mice fed a control diet; the decrease in minimum output (Fig 6C) was not statistically significant.

Learning and memory are complex events dependent on the electrochemical processes of long-term potentiation in the CA1 region of the hippocampus. The primary receptors involved in the processes of LTP are the NMDA receptors for induction and the increased insertion of AMPA receptors for its expression [28]. The HFD-fed group expressed a substantially reduced LTP (Fig 6D) as compared to controls (a 24.1% decrease; *p* < 0.01) (Fig 6E). These data showed that a HFD was responsible for eliciting deficits in hippocampal LTP.

## Discussion

In this experimental model of HFD-induced obesity, systemic insulin resistance—as indicated by the HOMA-IR values (Fig 1E)—developed at 8 weeks of HFD feeding. Insulin resistance plays a prominent role in the etiology of metabolic syndrome cluster of diseases [35]. Of note, we recognize that the control and the high-fat diet used in this study do not have identical micronutrient composition (as mentioned in Materials and Methods section). However, it is not likely that these differences may impact the impaired HFD-driven changes in synaptic plasticity in this study.

Some of the effects of HFD on liver function are underscored by a decreased activation of IRS-1 and increased activation of p38 and JNK, as well as the increased expression of NFκB associated with inflammatory responses (Fig 2). Both, impaired IRS-1/Akt pathway and inflammatory responses impinge on energy (mitochondrial) metabolism, reducing both hepatocyte basal respiration and oxidative phosphorylation, effects only partly ascribed to a decreased mitochondrial biogenesis. These data are in line with the notion that the pathogenesis of insulin resistance could be induced by accumulation of hepatic fat and impaired β-oxidation [6, 36] and the activation of inflammatory responses [37].

The expression of iNOS, downstream from NFκB activation, was increased over 10 fold in the hepatocytes of HFD-fed mice (Fig 2B), in agreement with the iNOS-induced nitrosative stress in the pathogenesis of insulin resistance in an obese mouse model [38] and by the higher distribution of iNOS in the fatty liver of a HFD-induced obesity model [39].

Although hepatocytes from HFD mice show a higher activation of JNK (Fig 2B), it is likely that this activation is the response to adipose-derived signals as shown in other obese models [40]. Activation of JNK by oxidative stress, fatty acids, and cytokines was enhanced in HFD-induced models of obesity and insulin resistance [41, 42]. In this regard, the JNK signaling pathway has been identified as a regulator of insulin signaling by either phosphorylating IRS at Ser^307^ or counteracting Akt-mediated inhibition of FoxO transcription or transcriptional repression of insulin in insulin-producing cells [29]. Hence, the cross- talk between JNK signaling and insulin signaling in this HFD-induced obesity model seems to be center-stage of insulin resistance. The deficient energy metabolism observed in the current study can be partly explained by endoplasmic reticulum stress (ER)-induced binding of JNK to the outer mitochondrial membrane protein Sab (SH3 homology associated BTK-binding protein): this leads to impaired mitochondrial respiration, which further sustains JNK activation [43, 44]. These findings may also be of significance because the insulin resistance observed in obese individuals is apparently caused by ER-mediated activation of JNK followed by phosphorylation of IRS-1 [45]. JNK translocation to the mitochondrion and impairment of energy metabolism has been reported in other experimental models [46]. Therefore, different pathways may account for the hepatocyte energy deficit observed in this study (Fig 3), mainly a direct interaction of JNK with the outer mitochondrial membrane and JNK-mediated inactivation of IRS-1.

The above factors contribute to different extent to the insulin resistance-mediated dysregulation of liver metabolism and establish a connection with insulin resistance in the brain: cytotoxic lipids, such as ceramide—formed in liver—can cross the blood-brain barrier and cause insulin resistance in the CNS [47].

Insulin signaling is responsible for glucose transport in the brain and control of energy homeostasis and is involved in the regulation of neuronal growth and synaptic plasticity [48, 49]. The HFD group showed a decreased Akt-dependent translocation of brain glucose transporters (GLUT4 and GLUT3) to the plasma membrane [50–52] and decreased activation of the IRS as well as the Ras/Raf/ERK signaling node (Figs 4 and 5). Both the IRS/PI3K route and the Ras/Raf/ERK signaling node are parallel signaling pathways downstream of the insulin receptor [9]. The Ras/Raf/ERK signaling node acquires further significance when considering the resulting induction of CREB (as seen in Fig 5), a transcription factor involved in learning and memory and modulator of the gene expression induced by BDNF [53]. CREB-dependent gene transcription is important for certain forms of synaptic plasticity and was reduced in a HFD rodent model [54]. An imbalance between insulin signaling and JNK signaling was also observed in brain as a function of age [55].

The impaired insulin signaling in the brain was expressed in terms of changes in synaptic strength and LTP: a substantial decrease in the former parameter indicated that the strength of synaptic connections was severely affected in the HFD group. The latter parameter, LTP, is usually considered critical for learning and memory and encompasses release of glutamate from the pre-synaptic terminal, activation of the NMDA receptor and depolarization followed by Ca^2+^ activation of signaling cascades [56]. This decrease in LTP was more pronounced than that reported on a triplet transgenic mouse model of Alzheimer's disease [28]. Several factors may contribute to the loss of synaptic plasticity in this HFD-induced obese model: (a) impaired insulin signaling (IRS and Ras/Raf/ERK signaling nodes) has profound effects on synaptic strength and LTP; (b) the decreased translocation of glucose transporters GLUT3/GLUT4 indicates reduced glucose uptake and impairment of metabolic homeostasis, and (c) reduced activation of the ERK/CREB pathway may affect LTP at a transcriptional level. As in a previous study [28], this research does not provide a causal relationship between decrease in glucose metabolism (e.g., reduced the concentration of GLUT3 and GLUT4 in the plasma membrane; Fig 4) and the high energy-demanding synaptic transmission (Fig 6). Despite this, other studies support the notion that HFD affects synaptic transmission, albeit in different experimental models: (a) a high-fat, refined sugar diet affected brain function via the regulation of neurotrophins, i.e., decreased the expression BDNF and downstream mRNA levels for CREB and synapsin I, and consequent impairment of spatial learning [57]. Of note, these changes were observed during a period of 2- to 24 months of high fat-, refined sugar feeding [57]. (b) A study on mice fed a HFD immediately after weaning and for 9–12 months showed behavioral deficits evaluated as step-down passive avoidance and contextual fear conditioning [16]. (c) Brain insulin resistance was observed only after 17 days HFD feeding and was manifested as impaired spatial working memory [12]. (d) An even shorter period (7 days) with a high-fat, high-fructose diet was associated with alterations of insulin signaling, reduction of dendritic arborization and decreased dendritic spine number in CA1, and increase in reactive astrocytes [58]. (e) Long-term HFD (20–32 weeks) induced peripheral insulin resistance and completely abolished the LTP in CA1 of hippocampus in a mouse model[18].

Despite some discrepancies between the experimental model in this study and that in those mentioned above [12, 16, 18, 57, 58], it may be surmised that a HFD (with or without refined sugars or fructose and from periods ranging from 1 week to 2 years) elicits alterations in brain function that are mostly associated with either a systemic- or brain insulin resistance. Data in this study unveil the effect of the HFD-induced systemic insulin resistance on liver and brain. Importantly, it is demonstrated that HFD could induce brain insulin signaling impairment and loss of synaptic strength and plasticity.

## Supporting Information

S1 Fig Glucose tolerance tests and liver weight. Mice were fed with 12-week HFD or normal diet and different parameters were monitored weekly or monthly. (A) Glucose tolerance tests of HFD group mice at 0, 4, 8, 12 weeks, n = 10 mice/group; (B) liver weight of Control group and HFD group mice ≥ 5 mice/group. Data presented as mean ± SD, *p < 0.05, **p < 0.01 *versus* control group.
(TIF)

S1 File Raw data. The meta-data including OCR, LTP, and all analysis.
(XLSX)
