# Supplementary figures and images for: High-Fat Diet Induces Hepatic Insulin Resistance and Impairment of Synaptic Plasticity
Source: PLoS One. 2015 May 29;10(5):e0128274. doi: 10.1371/journal.pone.0128274 (PMC4449222; doi:10.1371/journal.pone.0128274)

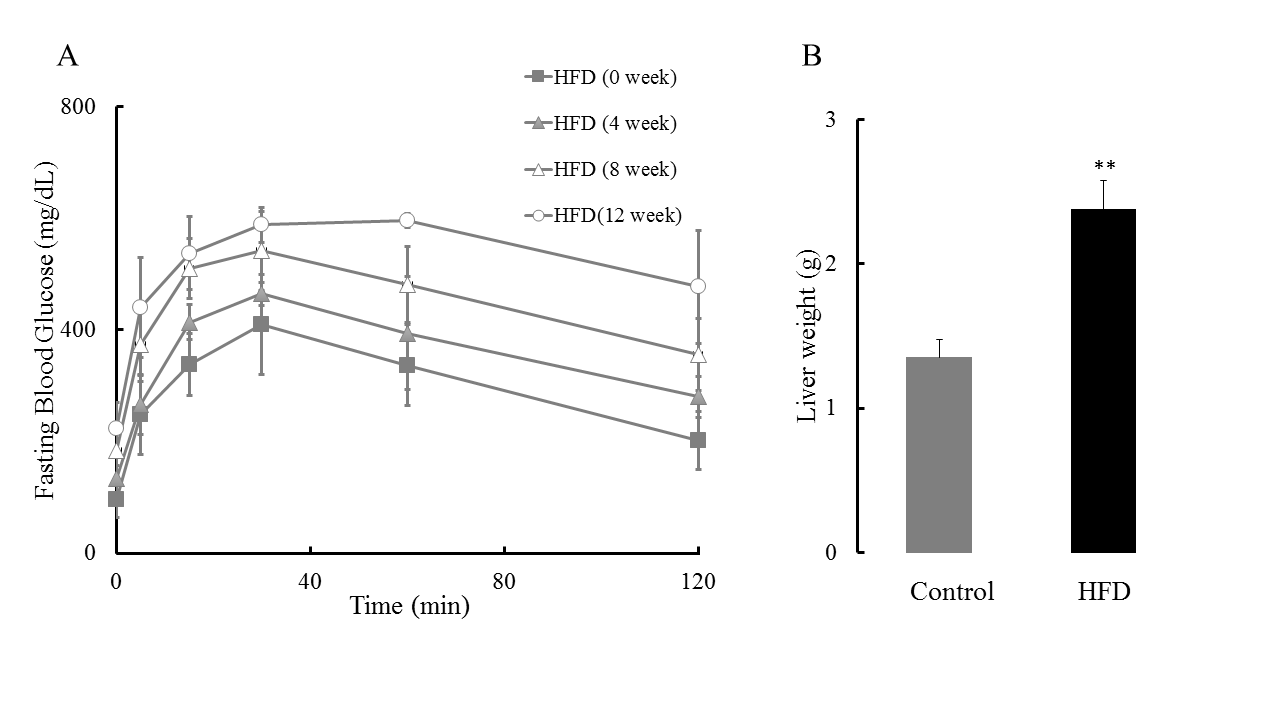

Supplement: S1 Fig — Mice were fed with 12-week HFD or normal diet and different parameters were monitored weekly or monthly. (A) Glucose tolerance tests of HFD group mice at 0, 4, 8, 12 weeks, n = 10 mice/group; (B) liver weight of Control group and HFD group mice ≥ 5 mice/group. Data presented as mean ± SD, *p < 0.05, **p < 0.01 versus control group. (TIF) [file pone.0128274.s001.tif]
